# Enhanced osteogenic potential of iPSC-derived mesenchymal progenitor cells following genome editing of GWAS variants in the RUNX1 gene

**DOI:** 10.1038/s41413-024-00369-x

**Published:** 2024-12-06

**Authors:** Nazir M. Khan, Andrea Wilderman, Jarred M. Kaiser, Archana Kamalakar, Steven L. Goudy, Justin Cotney, Hicham Drissi

**Affiliations:** 1 Department of Orthopaedics, Emory University School of Medicine, Atlanta, GA USA; 2 Atlanta VA Medical Center, Decatur, GA USA; 3 Department of Genetics and Genome Sciences, University of Connecticut, Farmington, CT USA; 4 Department of Otolaryngology, Emory University School of Medicine, Atlanta, GA USA

**Keywords:** Bone, Osteoporosis

## Abstract

Recent genome-wide association studies (GWAS) identified 518 significant loci associated with bone mineral density (BMD), including variants at the RUNX1 locus (rs13046645, rs2834676, and rs2834694). However, their regulatory impact on RUNX1 expression and bone formation remained unclear. This study utilized human induced pluripotent stem cells (iPSCs) differentiated into osteoblasts to investigate these variants’ regulatory roles. CRISPR/Cas9 was employed to generate mutant (Δ) iPSC lines lacking these loci at the RUNX1 locus. Deletion lines (Δ1 and Δ2) were created in iPSCs to assess the effects of removing regions containing these loci. Deletion lines exhibited enhanced osteogenic potential, with increased expression of osteogenic marker genes and Alizarin Red staining. Circularized chromosome conformation capture (4C-Seq) was utilized to analyze interactions between BMD-associated loci and the RUNX1 promoter during osteogenesis. Analysis revealed altered chromatin interactions with multiple gene promoters including RUNX1 isoform, as well as SETD4, a histone methyltransferase, indicating their regulatory influence. Interestingly, both deletion lines notably stimulated the expression of the long isoform of RUNX1, with more modest effects on the shorter isoform. Consistent upregulation of SETD4 and other predicted targets within the Δ2 deletion suggested its removal removed a regulatory hub constraining expression of multiple genes at this locus. In vivo experiments using a bone defect model in mice demonstrated increased bone regeneration with homozygous deletion of the Δ2 region. These findings indicate that BMD-associated variants within the RUNX1 locus regulate multiple effector genes involved in osteoblast commitment, providing valuable insights into genetic regulation of bone density and potential therapeutic targets.

## Introduction

Bone mineral density (BMD) is a strong surrogate measure of bone strength and a strong predictor of fracture risk.^1^ Fracture risk is higher in individuals with lower BMD.^2–4^ BMD is a complex trait which is regulated by various factors including behavioral, environmental, and genetic factors. Genetic factors contribute to 60%–80% risk to an individual with osteoporosis, which is primarily characterized by loss of BMD.^5^ As opposed to candidate gene approach, genome wide association studies (GWAS) are less biased method to comprehensive identify loci related to complex trait or disease. Recent GWAS using 426 824 individuals, identified 518 genome-wide significant loci associated with enhanced BMD (measured by heel ultrasound).^6^ This study replicated several known loci associated with BMD in prior GWAS and meta-analysis reports.^7,8^ Interestingly, all these genetic studies reported multiple variants associated with changes in BMD near the *RUNX1 gene*.^6–8^ We have pioneered the function of RUNX1 as regulator of bone homeostasis and using multiple conditional knockout mice studies, we established that RUNX1 activity can influence various components of skeletal development and repair.^9–12^

The reported BMD GWAS-variants at the *RUNX1* locus (rs13046645, rs2834676, and rs2834694) lie in intronic and intergenic regions, which raises the question how these noncoding variants affect the regulation of BMD? Although GWAS is an effective strategy to dissect the genetic basis of complex traits and diseases, it identifies only the sentinel SNPs (single nucleotide polymorphisms) with strongest association in a particular genomic locus, which may not be actual causal variants due to the presence of other SNPs in linkage disequilibrium (LD). Furthermore, most SNPs identified by GWAS across hundreds of diseases and phenotypes are non-coding.^13^ They have been shown to be enriched in putative regulatory elements such as such as enhancers in intronic and intergenic spaces across the genome.^14^ These findings indicate that impact of GWAS SNPs on phenotype occurs primarily via gene regulation potentially through alteration of transcription factor binding sites and enhancer-promoter interactions.^15,16^ However, the identification of the targets of such regulatory elements harboring GWAS-SNPs is challenging as the target genes might not be the nearest gene and located considerable distances away.^17,18^ Thus, while BMD variants have been located within and near the *RUNX1* gene it is unknown whether they regulate this gene. Based on the findings described above there is the distinct possibility that regulatory elements harboring BMD GWAS may regulate another effector gene at this locus thus affect the reported changes in BMD. Therefore, we set out to determine if putative regulatory sequences harboring BMD GWAS SNPs physically interact with *RUNX1* and functionally establish their role in skeletal differentiation and bone formation. We used human induced pluripotent stem cells (hiPSCs) differentiated to mesenchymal stromal cells (iMSCs) as an experimental model to first characterize three-dimensional chromatin contacts at this locus. We found that these regulatory sequences interact with multiple genes including *RUNX1* and *SETD4*, a histone methyltransferase gene. These results suggested that regulation of BMD at this locus may involve both *RUNX1* and other chromatin-related functions. To determine the functional requirements of these regulatory regions, mutant (Δ) iPSC lines were generated by deleting two distinct loci harboring these variants using CRISPR-Cas9 genome editing. We then derived iMSCs from both wild type and mutant *RUNX1* iPSC lines and determined their osteogenic commitment in vitro and bone formation in vivo using a calvarial defect model on SCID mice. Using these integrated approaches, we identified a novel regulatory hub at this locus that regulates multiple genes and influences osteogenic potential.

## Results

### 4C-sequencing at BMD GWAS locus showed interaction with multiple genes including RUNX1

**Fig. 1:**
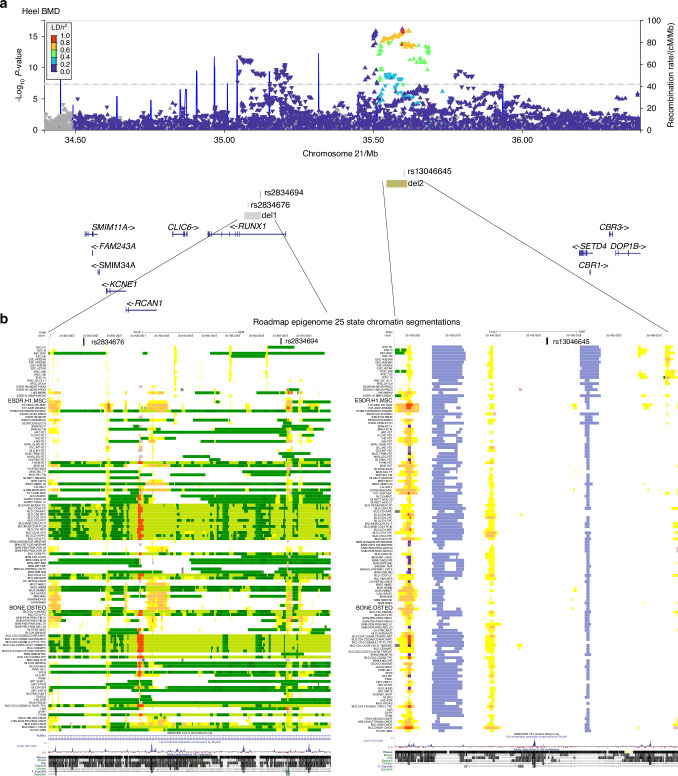
Two distinct loci are associated with heel bone mineral density near the *RUNX1* gene. **a** Locus Zoom plot of –Log_10_ *P*-values of individual tested variants for association with heel BMD as reported by Morris et al. ^6^ Colored triangles indicate linkage disequilibrium (LD) in European populations based on 1 000 G data relative to variant rs13046645. Lead variants reported by Morris et al at this locus are indicated by rs identifiers. Regions targeted for deletion in this study are indicated as del1(Δ1) and del2(Δ2); **b** Chromatin state segmentations from over one hundred cell and tissue types characterized by Roadmap Epigenome. Yellows and orange segments indicate putative regulatory elements based on chromatin signatures. Chromatin states for MSCs derived from H1 ESCs and osteoclasts are highlighted

**Fig. 2:**
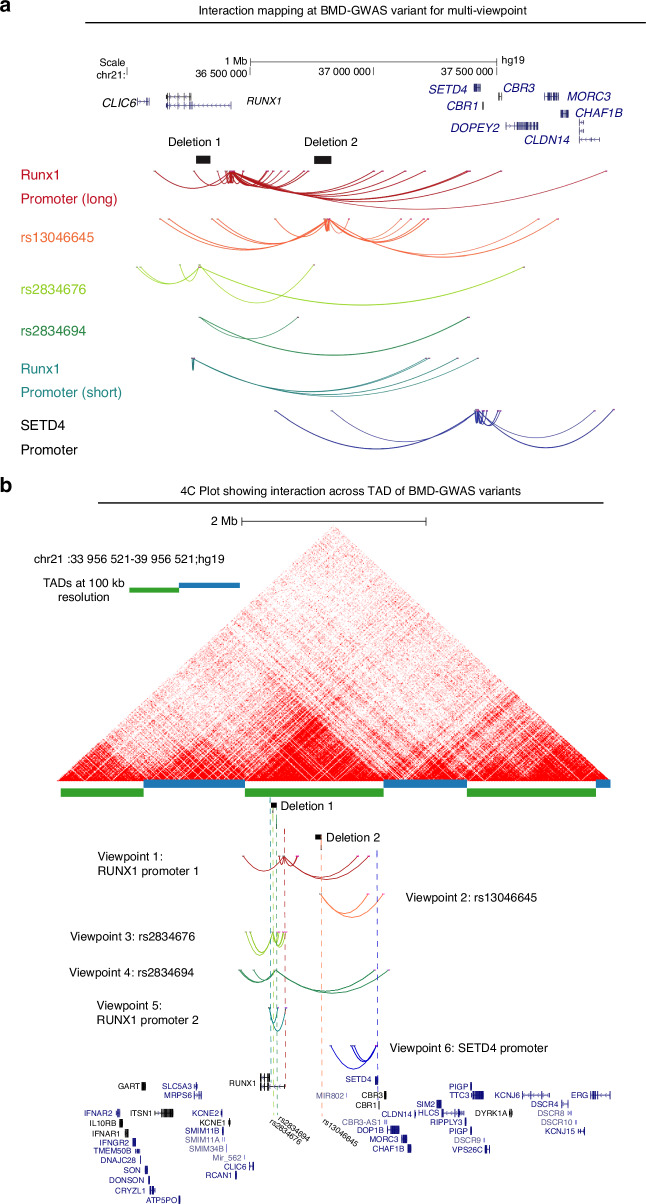
4C-sequencing at BMD GWAS locus showed interaction with multiple genes including RUNX1: **a** Interactions mapping at BMD associated variants by Capture C identified multiple significant interactions for each viewpoint. Promoters of both the long and short isoforms of RUNX1 exhibit long-range interactions with multiple genes including *SETD4* promoter; **b** 4C plot in relation to HiC data in wild type iMSCs. From top: publicly available HiC data from H1 hESC-derived MSCs processed via HiC-Pro and visualized using HiCExplorer as described in Methods. Topologically Associating Domains (TADs) that are determined at 100 kb resolution are depicted by blue and green alternating bars. Locations of the deletions are shown as black solid bars for reference, but the genomic sequences in those regions are present in WT-iPSC derived MSCs. Colored dotted lines represent the location of 4C viewpoints relative to the deletions, the SNPs and gene promoters. Viewpoint color assignments are consistent between the dotted line and arc interactions and full viewpoint label appear at left or at right. A simplified UCSC gene track at bottom contains the relevant *RUNX1* and *SETD4* isoforms as well as selected isoforms of surrounding genes

Given the complexities of assigning GWAS variants to target genes based solely on linear genomic distance, we wanted to test for physical interactions of each locus associated with BMD as well as the RUNX1 promoter and promoters of nearby genes. We processed publicly available HiC data for H1-ESC differentiated to iMSCs and observed a large topologically associated domain (TAD) that encompassed the RUNX1 gene and extended over 1 megabase to *CBR1*. Inspection of each BMD GWAS variant locus revealed potential regulatory sequences. Variants rs2834676 and rs2834694 were within an intron of the long isoform of RUNX1 based on GENCODE (v44) annotation. In particular, there were putative enhancers flanking both variants that are active in MSCs derived from H1 ESCs as well as chondrocytes derived from these MSCs (Fig. 1a). The third variant, rs13046645, was located near a deeply conserved noncoding sequence, a putative enhancer active in most cell types, and flanked by two regions that have repressive chromatin signatures (Fig. 1b). Having demonstrated potential for high-level chromatin domain that encompass multiple BMD GWAS variants we set out to identify higher resolution interactions within the locus. We used 4C-sequencing in iMSCs to identify the targets of BMD GWAS variants at the *RUNX1* locus. We chose primers for five distinct viewpoints to selectively amplify from circularized chromosome conformation libraries (Fig. S1). Analysis with PeakC^19^ identified multiple significant interactions for each viewpoint (Fig. 2a). We found that promoters of both the long and short isoforms of RUNX1 form long-range interactions with multiple genes at the opposite end of the TAD. Both promoters interacted with the promoter of *SETD4*. Using SETD4 as a viewpoint revealed reciprocal interactions to the long isoform promoter of RUNX1 and to the region at the center of the TAD harboring variant rs13046645 (Fig. 2b). Subsequently using rs301406645 as a viewpoint we observed extensive interactions across the TAD suggesting promiscuous or dynamic interactions for this locus. Both viewpoints near rs2834676 and rs283694 revealed similar interactions as expected given their relatively close proximity on the linear genome (Fig. 2b). These loci interacted with gene promoters at the opposite end of the TAD but also with the locus harboring rs301406645. These findings suggest that each of these BMD-associated variants may implicate regulatory regions that control expression not only of *RUNX1* but also *SETD4* and potentially other genes located quite far away. To begin to understand this long-range gene regulation we sought to remove these putative regulatory loci and measure their effects on gene expression on multiple putative targets in the region.

### CRISPR/Cas9 mediated genome editing for deletion of *RUNX1* intronic regions containing BMD GWAS variants

**Fig. 3:**
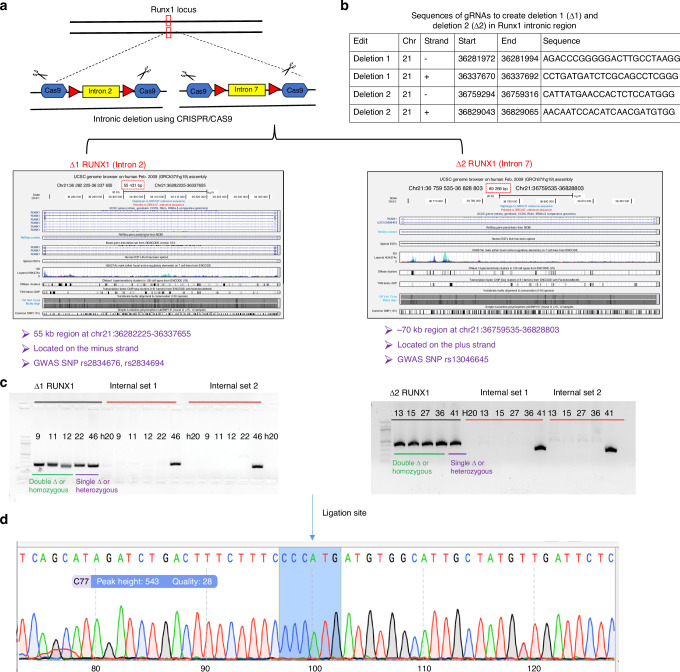
CRISPR/Cas9 mediated genome editing of hiPSC at BMD-GWAS loci near *RUNX1* gene promoter. **a** Schematic diagram of a strategy to delete GWAS locus at *RUNX1* intronic region. Deletion 1 (Δ1) was 55 kb region in intron 2 at chr21:36282225-36337655 containing GWAS SNP rs2834676. Deletion 2 (Δ2) was of 70 kb region in intron 7 at chr21:36759535-36828803 harboring GWAS SNP rs2834694; **b** Sequences of gRNAs used to create Deletion 1 (Δ1) and Deletion 2 (Δ2) in *RUNX1* region. These guide RNAs were shown to flank the region to be deleted; **c** Gel image of PCR showing deletion of RUNX1 intronic region at single or double allele. The PCR is done using a primer located upstream of the upstream cut site and downstream of the downstream cut site. The internal primers are primers located within region removed; **d** Sanger sequencing to confirm deletion mutant 2 (Δ2) in *RUNX1* region in BD1-4-iPSCs. Sequence across the cut site indicates the conical ligation of the two cut sequences. The double stranded break is 3 base pairs up from the NGG and sequencing of the clones show the fusion of these at that location. The conical fusion read as CCCATGATGTGG. The underlined sequence indicates CRISPR guide site

As noted above, prior genetic studies identified 3 SNPs in *RUNX1* gene (rs13046645, rs2834676, and rs2834694) associated with trabecular vBMD in older men.^6–8^ Since it is unclear whether these variants are the causative variants and the predicted effect sizes are quite small from these studies, we sought to remove the regulatory loci completely using genome-editing approach to increase the likelihood of a measurable effect. To determine the regions, we combined linkage disequilibrium data for European Ancestry populations from the 1 000 Genomes Project^20^ with mammalian sequence conservation, and chromatin state annotations from relevant cell types profiled by Roadmap Epigenome including H1 derived MSCs and osteoblasts^21^ (Fig. 1b). Based on these genetic and functional annotations, we identified two linkage disequilibrium blocks for ablation of ~55 kb and ~70 kb at positions chr21:36282225-36337655 and chr21:36759535-36828803, respectively. These were denoted as deletion mutant 1 (Δ1-RUNX1-iPSCs), encompassing the SNPs rs2834676 and rs2834694, and deletion mutant 2 (Δ2- RUNX1-iPSCs), encompassing SNP rs13046645, respectively (Fig. 3a).

We used skin fibroblast derived hiPSC (BD1-4) to generate the deletion mutant using CRISPR/Cas9 based editing approach as described previously.^22^ Briefly we used BD1-4 hiPSCs to transiently transfect with plasmid expression Cas9 and pairs of guide RNAs targeting the flanking DNA to each of these regions. The sequences of gRNAs to create deletion 1 (Δ1) and deletion 2 (Δ2) in *RUNX1* region were shown in Fig. 3b. With Sendai virus-based targeting strategy, we readily obtained multiple clones from these iPSC lines with deletion at one allele (heterozygous deletion) or at both alleles (homozygous deletion) for each deletion sites. These selected iPSC clones were further expanded and characterized to confirm the targeted deletion by PCR (polymerase chain reaction) amplification (Fig. 3c) and Sanger sequencing (Fig. 3d). We generated both homozygous and heterozygous mutant clones lacking both and single allele respectively. Multiple clones for each deletion were generated Δ1-*RUNX1*-Homozygous (#9, #11), Δ1-*RUNX1*-Heterozygous (#46), Δ2-*RUNX1*-Homozygous (#13, #27), Δ1-*RUNX1*-Heterozygous (#42) (Fig. 3c).

### Characterization of mutant iPSCs (Δ-RUNX1-iPSC) for pluripotency markers

**Fig. 4:**
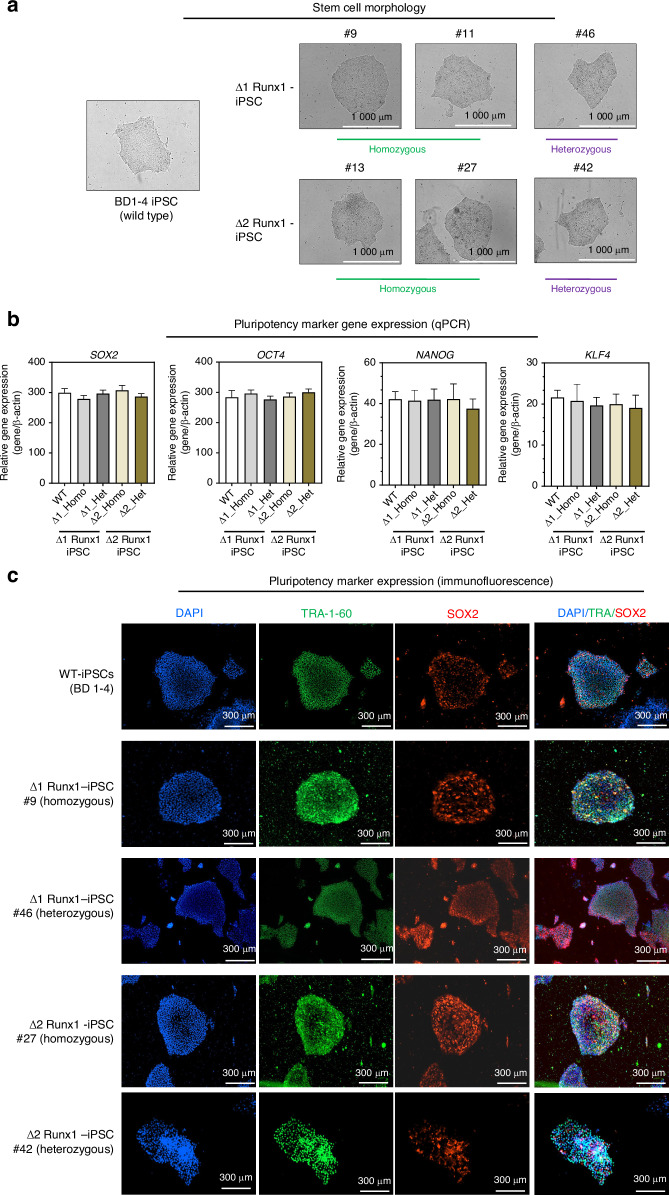
Characterization of mutant iPSCs (Δ-*RUNX1*-iPSC) for pluripotency markers: **a** Morphology of the wild type BD1-4 hiPSCs, deletion mutant clone- Δ1-*RUNX1*-iPSC (#9, #11, #46) and Δ2-*RUNX1*-iPSC (#13, #27, #42) colonies in monolayer culture on a 0.1% Geltrex coated plate; Image displays representative experiment (*n* = 3); **b** Pluripotency for iPSC colonies showing expression of stemness genes. RT-qPCR analyses showed induced expression of canonical stemness genes *SOX2, OCT4, NANOG* and *KLF4* in wild type and deletion mutants (Δ1 and Δ2-*RUNX1*-iPSC) colonies. β-actin served as the housekeeping gene and internal control. Results from one representative experiment (*n* = 3). Represented gene expression data is relative to MSCs derived from respective iPSC cells; *P ≤ 0.01, as compared to their respective iMSCs; **c** Immunofluorescence staining of pluripotency markers in wild type (BD1-4), homozygous Δ1-*RUNX1*-iPSC (#9), heterozygous Δ1-*RUNX1*-iPSC (#46), and homozygous Δ2-*RUNX1*-iPSC (#27), heterozygous Δ2-*RUNX1*-iPSC (#42) showed expression of surface TRA-1-60 and SOX2 antigens in these colonies. DAPI is used as nuclear counterstain showing blue nuclei. Scale bar, 100 μm. Image displays representative experiment (*n* = 3)

To determine whether deletion of BMD GWAS variants at *RUNX1* intronic region had any effect on the expression of pluripotency markers of generated clones, we performed extensive characterization of mutant clones using morphological, gene expression, and stemness marker expression analysis. We used multiple clones of the homozygous and heterozygous deletion at each locus (Δ1 and Δ2) and compared their pluripotency markers, progenitor properties and osteogenic potential with wild-type parental clone. As shown in Fig. 4a, morphological analysis showed undifferentiated pluripotent stem cell phenotype of both Δ1-iPSC and Δ2-iPSC clones (Fig. 4a). Stemness characteristics of these iPSC clones was evaluated via qPCR assessment of key pluripotency marker genes. The mRNA copy number of *SOX2*, *OCT4, NANOG* and *KLF4* were comparable in wild type and deletion mutants (Δ1-iPSCs and Δ2-iPSCs) indicating a similar level of stemness identity between these iPSCs suggesting that deletion of non-coding GWAS region at *RUNX1* gene did not affect the pluripotency state of deletion mutants (Fig. 4b). Pluripotency marker expression was also confirmed using immunofluorescence staining in wild type and both deletion mutants and our results demonstrated that cell colonies (homozygous and heterozygous) from both Δ1-iPSCs and Δ2-iPSCs showed positive expression of SOX2 and TRA-1-60 proteins (Fig. 4c). Our assessment of expression of pluripotency markers was a technical validation to ensure the integrity of our iPSC model and verify that the deletion mutants exhibited comparable pluripotency marker profiles to the wild type iPSCs, thereby isolating the effects of the targeted genomic deletions.

### Derivation of mesenchymal stromal cells (iMSCs) from mutant iPSCs (Δ-RUNX1-iMSC) and characterization of mesenchymal nature in vitro

**Fig. 5:**
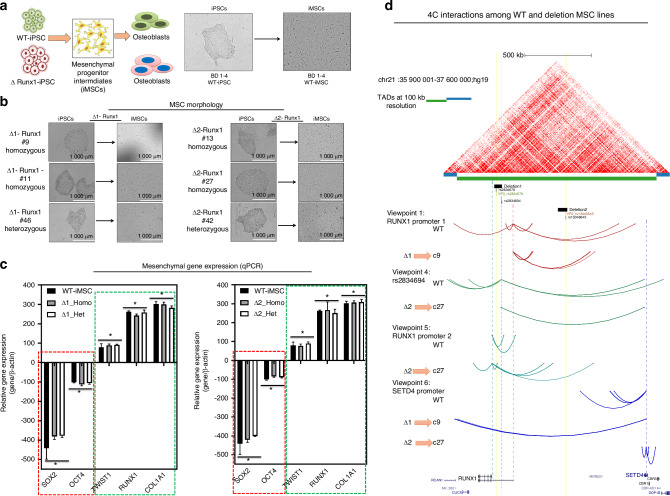
Derivation of mesenchymal stromal cells (iMSCs) from mutant iPSCs (Δ-*RUNX1*-iMSC) and characterization of their mesenchymal feature: **a** Derivation of mesenchymal progenitors as intermediate populations from wild type and mutant iPSCs using direct plating methods; **b** The morphology of the iMSC-like cells (iPSC-MSC) derived from Δ1- and Δ2-*RUNX1*-iPSCs showing elongated spindle shaped cells. Representative images are shown for iMSCs at passage 5-8. Scale bar, 100 μm; **c** Gene expression analyses by qPCR showing significant suppression of pluripotent markers *OCT4*, and *SOX2* and induction of mesenchymal genes *TWIST1, COL1A1*, and *RUNX1* in wild type (BD1-4) and Δ1- and Δ2-RUNX1-iMSCs relative to their parental iPSCs. β-actin served as the housekeeping gene and internal control. Expression data is represented as fold change relative to respective parental iPSCs. *P ≤ 0.01, as compared to their respective iPSCs; **d** Comparison of 4C interactions among WT-iMSC and deletion MSC lines (Δ1 and Δ2). From top: publicly available HiC data from H1 hESC-derived MSCs processed via HiC-Pro and visualized using HiCExplorer as described in Methods. TADs that are determined at 100 kb resolution are depicted by blue and green alternating bars, with the RUNX1 TAD in green. The location of Deletions 1 and 2 are shown as a black horizontal bar for reference. Colored dotted lines represent the location of 4C viewpoints relative to the deletions, the SNPs and gene promoters. Viewpoint color assignments are consistent between the dotted line and arc interactions and full viewpoint label appear at left as well as the cell line of origin. Viewpoints 2 and 3 are not shown because significant peaks were only detected in WT. For the remaining viewpoints, interaction is only shown for lines that had significant interactions within the RUNX1 TAD. A simplified UCSC gene track at bottom contains the relevant RUNX1 and SETD4 isoforms as well as selected isoforms of surrounding genes

Differentiation of iPSCs into osteoblast requires mesodermal induction,^23,24^ therefore, we derived mesenchymal stromal cells as intermediate populations from wild type and mutant iPSCs. We used our established direct plating methods in the presence of serum and human recombinant bFGF for differentiation of hiPSCs into mesenchymal stromal cells termed as iMSCs^25–27^ (Fig. 5a). MSCs derived from wild type iPSCs and deletion mutants Δ1-RUNX1-iPSCs (termed as Δ1-RUNX1-iMSCs) and Δ2-RUNX1-iPSCs (termed as Δ2-RUNX1-iMSCs) exhibited similar phenotypic characteristics of spindle-shaped and elongated morphology (Fig. 5b). Furthermore, we did not observe any morphological difference between homozygous and heterozygous deletion mutants as MSCs generated from these iPSC lines exhibited uniform fibroblast-like morphology (Fig. 5b).

We further characterized these iMSCs for mesenchymal and pluripotency markers using gene expression and flowcytometric analyses to determine whether the iPSCs had successfully differentiated into iMSCs. Our qPCR analysis using panel of mesenchymal markers showed that these iMSCs robustly expressed *TWIST1*, *COL1A1* and *RUNX1* genes (Fig. 5c). Moreover, these cells also maintained negative expression of stemness marker genes such as *SOX2* and *OCT4* (Fig. 5c). Interestingly, our analysis did not find any significant difference between wild type iMSCs and deletion mutant iMSCs (Δ1 and Δ2) for the expression of mesenchymal marker genes indicating identical mesenchymal nature (Fig. 5c). Our immunophenotypic analyses indicated that these iMSCs express a range of surface markers typical of skeletal osteolineage cells, including CD44, CD73, CD90, CD105, and CD166 (Fig. S2). Furthermore, all these iMSCs showed little to no expression of the hematopoietic marker CD45, and the endothelial marker CD31 (Fig. S2). Our comparative analysis showed that deletion of non-coding region at *RUNX1* GWAS loci did not affect the mesenchymal characteristics and both homozygous and heterozygous deletion of Δ1 and Δ2 mutants iMSCs showed identical immunophenotype of wild type.

When we performed 4C-Seq in iMSCs derived from these deletion clones (Δ1 and Δ2) with the viewpoints that were not ablated we found some surprising effects on local chromatin folding. Deletion 1 ablated some of the longer-range interactions of the long-isoform RUNX1 promoter and instead allowed for very long interactions for the SETD4 promoter (Fig. 5d). Deletion 2 resulted in increased interactions for the short isoform promoter of *RUNX1* and had the opposite effect on the *SETD4* promoter. These findings suggested that while iMSC generation was unaffected there were substantial changes in 3D chromatin organization. Thus, we wondered if upon further differentiation these changes could have impacts on gene expression and influence osteogenic potential.

### Deletion mutant iMSCs (Δ1-*RUNX1* and Δ2-*RUNX1*) exhibited enhanced osteogenic potential in vitro

**Fig. 6:**
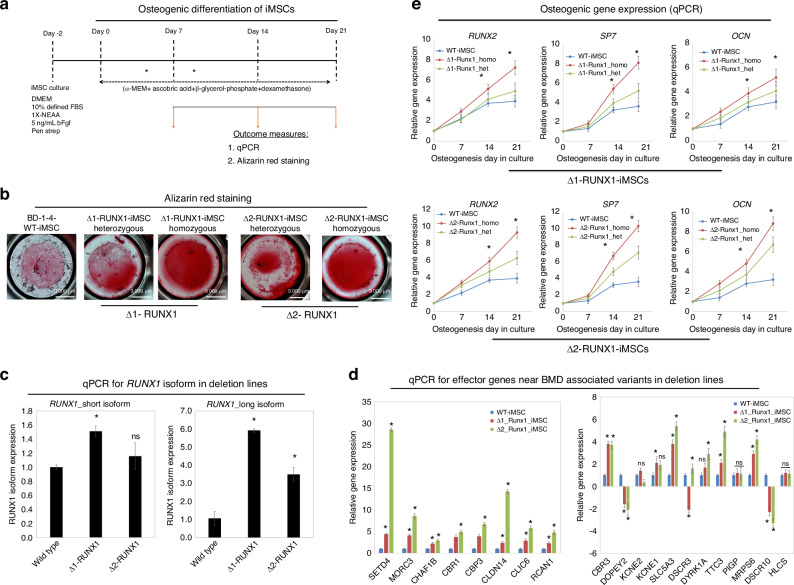
Deletion mutant iMSCs (Δ1-*RUNX1*and Δ2-*RUNX1*) exhibited enhanced osteogenic potential in vitro: **a** Schematic showing treatment conditions for in vitro osteogenic differentiation of wild type and Δ1- and Δ2-*RUNX1*-iMSCs using osteogenic medium consisting of alpha-MEM supplemented with 1 mmol/L sodium pyruvate, 0.1 μmol/L dexamethasone, 50 µg/mL ascorbic acid 2-phosphate, 10 mmol/L β-glycerophosphate, 10% FBS and 1X penicillin/streptomycin; **b** Osteogenic differentiation was shown by Alizarin Red staining of cultures in osteogenic media for 21 days. Alizarin Red staining revealed the mineralization of matrix showing osteoblast differentiation. Mineralization indicating enhanced osteoblast differentiation in homozygous deletion Δ1 and Δ2 as compared to wild type culture. Scale bar: 100 µm; **c** qPCR analysis for identifying the changes in expression of long and short isoform of RUNX1 in deletion mutants. β-actin served as the housekeeping gene and internal control. Values represent fold induction (Mean ± SD) relative to wild type iMSCs. *P ≤ 0.01 indicate values are statistically different in Δ-RUNX1-iMSCs as compared to their wild type control iMSCs; **d** qPCR analyssi of identified effector genes in wild type and deletion mutant-iMSCs (Δ1-*RUNX1* and Δ2-*RUNX1*). β-actin served as the housekeeping gene and internal control. Values represent fold induction (Mean ± SD) relative to wild type iMSCs from three replicate. *P ≤ 0.01 indicate values are statistically different in Δ-RUNX1-iMSCs as compared to their wild type control iMSCs; **e** Quantitative PCR analyses showing expression of key osteogenic marker genes *RUNX2, SP7* and *OCN* in Day 7, 14, and 21 osteogenic differentiations in wild type (BD1-4) and Δ1-RUNX1-homozygous (#9), Δ1-RUNX1-heterozygous (#46), Δ2-RUNX1-homozygous (#27) and Δ2-RUNX1-heterzygous (#42). β-actin served as the housekeeping gene and internal control. Values represent fold induction (Mean ± SD) relative to control iMSCs (Day 0) from three replicate. *P ≤ 0.01 indicate values are statistically different in Δ-RUNX1-iMSCs as compared to their wild type control iMSCs at each timepoint

To determine the functional role of these non-coding sequences harboring GWAS variants for bone formation, we performed an in vitro osteoblast differentiation assay using iMSCs from wild type and each of the *RUNX1* deletion mutants. The differentiation was performed by culturing these iMSCs in osteogenic medium for 21 days (Fig. 6a). On day 21, the cells were stained for mineralization using Alizarin Red S stain (Fig. 6b). Compared to the growth media, osteogenic media induced mineralization in wild type BD1-4 iMSCs at day 21 culture. Interestingly, both deletion mutant iMSCs (Δ1- and Δ2-RUNX1) exhibited significantly increased mineralization in a dose-dependent manner (Fig. 6b). Our data clearly demonstrated significantly increased mineralization in homozygous deletion mutants compared to heterozygous deletion mutants and wild-type iMSCs (Fig. 6b). The effects we observed were consistent with copy number of each region. The most pronounced osteogenic effects were observed in mutant lines lacking both regions on both alleles. This contrasts with modest effects in mutant lines lacking a single region on a single allele (Fig. 6b). These data suggest dosage of each noncoding region influences osteogenic differentiation potential and indicating a potential mechanism of how these BMD-associated variants impact bone formation.

To determine if these deletions impact expression of any of the genes in this locus we first examined expression of potential direct targets identified from the 4C data. When we performed qPCR for each isoform of *RUNX1* we found strong, significant increases in expression of the long isoform in each deletion line (Fig. 6c). More modest effects were observed for the short isoform with only Δ1 reaching statistical significance. As we expanded our analysis across the locus, we found most genes in the vicinity were significantly upregulated in both deletions. The largest effects were observed for *SETD4* with nearly 30-fold upregulation in the Δ2 line (Fig. 6d). These findings suggest these deletions cause significant changes in chromatin organization over nearly ~3 Mb resulting in a largely active domain increasing expression of most genes within.

We next determined the gene expression of osteogenic markers in both wild type and RUNX1 deletion mutant-iMSCs. Our qPCR analysis showed that wild-type iMSCs treated with osteogenic media for 7, 14, and 21 days exhibited increased expression of classical osteogenic markers including *RUNX2, SP7* and *OCN* in a time-dependent manner (Fig. 6e). Consistent with Alizarin red staining data, expression of these osteogenic genes was significantly higher in homozygous deletion mutants at both locus (Δ1- and Δ2-RUNX1-iMSCs) compared to heterozygous clones and wild types iMSCs at 14 or 21 days of osteogenic differentiation (Fig. 6e). However, no significant differences in expression of these key osteogenic genes were observed between iMSCs derived from wild type and deletion mutants at day 7 of osteogenic differentiation (Fig. 6e). These data indicate that non-coding regions harboring BMD GWAS loci near *RUNX1* regulate osteogenic differentiation and bone formation. These regions likely contain regulatory sequences which control the key steps involved in osteoblast differentiation and maturation.

### Deletion mutant 2 (Δ2-*RUNX1*-iMSCs) showed enhanced bone formation and repair of cranial defects in vivo

Since iMSCs derived from *RUNX1* deletion mutants showed an enhanced osteogenic commitment and differentiation in vitro, we next assessed whether administration of these mutant iMSCs showed similar effect in vivo and induce bone formation and repair of cranial bone defects in mice. To determine the regenerative capacity of the iMSCs derived from wild type and Δ-RUNX1 at both locus (Δ-1 or Δ-2), we used a parietal bone defect model of sub-critical-size (2.3 mm) in mice and determined bone regeneration by these iMSCs. To prevent rejection of human graft (iMSCs), we used immune deficient NOD-SCID (*n* = 10/group; total 30 mice).

Administration of MSCs alone to critically sized calvarial defects has not successfully regenerated bone due to low cell survival compared to when delivered in a delivery vehicle or scaffold.^28^ Therefore, we decided to encapsulate iMSCs in a synthetic hydrogel as a carrier for sustained release to the surgery site for the regeneration of calvarial bone. To this end, we used our established PEG (poly ethylene glycol) hydrogel to encapsulate these iMSCs immediately before implantation to defect site. Our group have previously demonstrated the use of this hydrogel with cranial neural crest for the repair of cranial bone defects in vivo.^29^ These hydrogels have been used extensively for targeted protein delivery because they are safe, elicit minimal inflammation, and have tunable properties.^30^ PEG hydrogels are made of four-arm PEG macromer that are end-functionalized by maleimide moieties (PEG-4MAL) to improve ligand binding and cross-linking. The iMSCs derived from wild type and deletion mutants were encapsulated in 4% PEG-4MAL hydrogel.

**Fig. 7:**
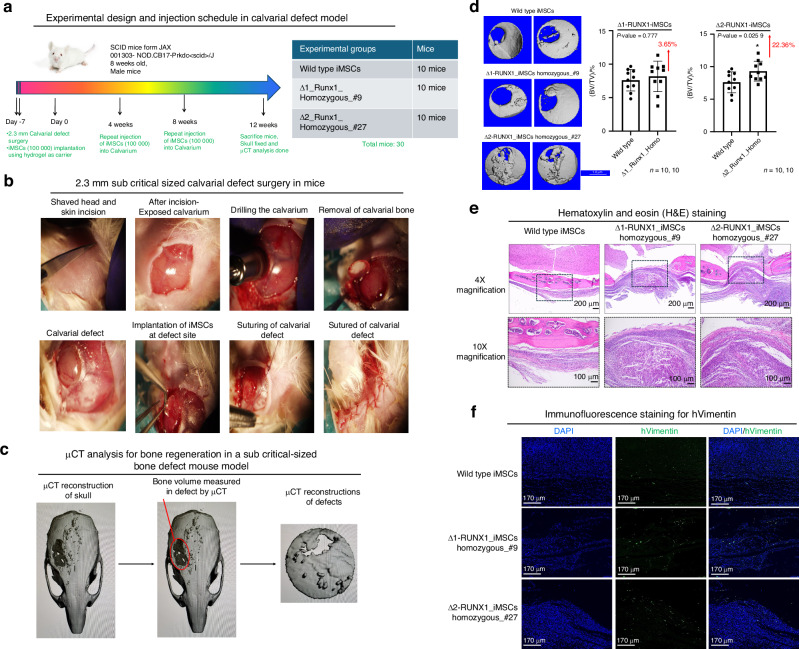
Deletion mutant 2 (Δ2-*RUNX1*-iMSCs) showed enhanced bone formation and repair of cranial defects in vivo: **a** Study design of calvarial defect in 8-week-old NOD-SCID mice. Wild type (BD-1-4)-iMSCs, homozygous deletion mutant Δ1-iMSCs (#9) and Δ2-iMSCs (#46) were implanted in defect site at 3 different time during surgery (day 0), and week 4 and week 8; **b** Surgical procedure involved in creation of 2.3 mm sub-critical-sized defects in the parietal bones of 8-week-old mice and delivery of iMSCs encapsulated with 4% PEG-MAL hydrogels; **c** Quantification of regenerated bone volume within the defect site at 12-week post surgery and μCT reconstructions of skull and defect size are shown; **d** Quantification of differences in regenerated bone volume (%BV/TV) within the defect and compared them between wildtype and Δ1-iMSCs (#9) and Δ2-iMSCs (#46) by μCT analysis. μCT reconstructions of defects in each experimental group were shown. Data are presented as mean ± SD with *P*-values reported. *P* ≤ 0.01 indicate values are statistically different in Δ-RUNX2-iMSCs as compared to their wild type control iMSCs. *n* = 10 mice per group as shown in Figure D. Data were analyzed by paired *t*-Test using Graph-pad Prism (V9.0); **e** Representative sections of the calvarial defect area on mice skulls from all three experimental groups were stained with hematoxylin and eosin (H&E) staining. Dashed boxed areas in the upper panel are shown at a higher magnification (10X) in the bottom panel for each sample; **f** Immunofluorescence staining of implanted iMSCs for detection of human vimentin to distinguish implanted human cells from host mice cells. Nuclear DNA was labeled with DAPI. Scale bars, 170 μm

To test the regenerative capacity of the iMSCs encapsulated in PEG-4MAL hydrogel, we implanted them in sub-critical-sized parietal bone defects (2.3 mm) in mice (Fig. 7b). The volume of bone regenerated by the Δ1-RUNX1-iMSCs and Δ2-RUNX1-iMSC was measured and compared to wild type iMSCs. To standardize the number of iMSCs injections required for the repair of calvarial defects, we initially performed a pilot experiment. We tested different injection schedules and assessed the efficacy of single vs. multiple injections for bone regeneration using µCT analysis. In multi-injection schedule, additional injection of iMSCs were performed transcutaneously at week 4, and week 8 (Fig. 7a). The pilot data showed marginal improvement in bone regeneration with a single iMSCs injection, while two injections increased the regenerated bone volume but not did not reach statistical significance. Interestingly, a three-injection schedule resulted in significant bone regeneration for calvarial defect repair (Fig. S3). Based on these findings, we adopted the three-injection schedule of hydrogel encapsulated iMSCs and compare the efficacy of different iMSC clones for bone repair at 12 weeks by quantifying the differences in bone volume (BV) within the cranial defect using μCT analysis (Fig. 7c). μCT reconstruction of defects demonstrated that in the absence of iMSCs, hydrogel control did not regenerate bone as demonstrated by lack of significant difference in bone volume between the hydrogel-control group and the empty defect group. These findings indicate that hydrogel scaffold did not contribute to bone regeneration in the mice calvarium (Fig. S4).

Delivery of hydrogels encapsulating wild-type iMSCs demonstrated noticeable bone regeneration, however, it did not show the closure of cranial defect (Fig. 7d). Interestingly iMSCs derived from homozygous RUNX1 deletion mutants at locus 2 (Δ2-RUNX1-iMSCs) showed 22.3% enhanced bone formation and significantly improved the closure of the cranial defect (Fig. 7d). Moreover, Δ1-RUNX1-iMSCs also showed increased bone regeneration than wild type iMSCs, however, it did not reach statistical significance (Fig. 7d). These results were in line with our in vitro osteogenesis data showing enhanced osteogenic potential of Δ2-RUNX1-iMSCs as compared to wild type iMSCs. To support the radiographic findings, we next performed histological analysis of the mouse skulls to verify the engraftment of implanted cells within the calvarial bone. We performed paraffin-embedded sectioning of the calvarial bone and conducted hematoxylin and eosin (H&E) staining to visualize the cellular structure and distribution at the repair site. The staining confirmed bone regeneration at the defect site, with enhanced bone repair observed in mice injected with Δ2_RUNX1-iMSCs compared to those injected with wild-type iMSCs or Δ1_RUNX1-iMSCs (Fig. 7e). Additionally, to identify the presence of human cells at the repair sites, we performed immunofluorescence staining using an anti-human vimentin antibody. Our results demonstrated the presence of immunoreactive human cells in calvarial defect area (Fig. 7f). However, not all cells showed immunoreactivity with human vimentin antibody (Fig. 7f). Our interpretation is that human iMSCs did not persist in the mice calvarial defect up to 12 weeks. Together, these findings suggest that deletion of non-coding sequences harboring BMD-associated variants near *RUNX1* stimulate signaling pathways which may induce osteogenic differentiation of host cells leading to enhanced osteoblast commitment for the repair of cranial bone defects. These results suggest that Δ2-RUNX1 deletion mutant may affect the regulatory sequences which control the expression of osteogenic genes. These regulatory sequences at *RUNX1* GWAS locus 2 may show long-range interaction with signaling genes involved in osteogenic differentiation pathways and thus regulate the osteogenesis and bone formation.

## Discussion

One of the main goals of human genetics research is to gain insights into the genetic factors contributing to complex diseases, particularly delving into the molecular mechanisms through which common DNA variants influence the etiology of diseases. The majority of GWAS implicate non-coding variants that are far from genes, leading to challenges in deciphering their mode of action and accurately identifying the intended target gene.^14^ Accumulating evidence suggests that disease-associated variants disrupt the functionality of cis-acting regulatory elements, such as enhancers, which in turn affect the expression of specific genes that serve as functional targets for these elements.^17^ However, the challenge persists in pinpointing the true functional targets of regulatory elements, given that these regulated elements can be situated kilobases (kb) away from their designated target gene(s).^18^ In this study, we used high-resolution promoter interaction maps to uncover target genes associated with GWAS findings. Here, we first examined chromatin interactions in human iMSCs to determine the true target (s) of GWAS variants at *RUNX1* region for changes in BMD and bone formation. By using 4C sequencing with viewpoints from several regions within this larger locus in wild-type cells, we were able to map all 3 BMD-associated variants and discover a list of novel genes that they physically interact. In addition to the *RUNX1* gene which initially captured our interest, our analysis also suggested *SETD4* might play a role in modulation of BMD.

In this study, we used hiPSCs derived mesenchymal stromal cells (iMSCs), a highly relevant cellular model for BMD and osteoporosis. Use of hiPSCs allowed us to interrogate a specific regulatory region by performing a locus specific deletions using CRISPR/Cas9 mediated genome editing approach. In this study, we deleted two specific regions of 55 Kb and ~70 Kb encompassing the BMD associated variants at the *RUNX1* gene locus to identify the effector target gene(s) by long-range interaction mapping analysis. Δ1-RUNX1-iPSCs encompass SNPs rs2834676 and rs2834694 within an intron 2 of the long isoform of RUNX1 which possessed potential regulatory sequences. Both the variants present in Δ1-RUNX1 were flanked by putative enhancer suggesting potential regulatory role, however, our deletion mutant Δ1 does not allow us to discern the individual contributions of rs2834676 and rs2834694 to BMD regulation. Future studies are needed to create separate iPSC lines with individual deletions of rs2834676 and rs2834694 using CRISPR-Cas9 genome editing. This will help elucidating the specific functional roles of each SNP on gene expression, chromatin interactions, and osteogenic potential, providing a more detailed understanding of their individual contributions to BMD and skeletal development.

Recent long-range interaction mapping studies suggest that ~10% of promoter interactions are confined to the nearest genes, whereas more than 90% of accessible disease variants establish interactions with genes situated at a considerable distance.^16^ Our analysis identified multiple gene targets of BMD-associated loci near *RUNX*1. These effector genes included *SETD4, MORC3, CLDN14, SLC5A3, TTC3, DYRK1A, CLIC6* and *RCAN1*. Among these *SETD4* showed robust expression in deletion mutant 2 (Δ2-RUNX1-iMSCs, #27) and formed long-range interactions to BMD-associated loci, making it a candidate functional target of the GWAS associations. Most intriguingly, this gene is not well known to bone community as available literature did not show any bone-related phenotype for *SETD4*. Although our studies have not probed the mechanistic basis for the regulation of osteoblastogenesis by *SETD4*, we suggest epigenetic roles of *SETD4* could affect osteoblast differentiation. *SETD4* has been reported to be a histone-lysine N-methyltransferase that catalyzes methylation of lysine 20 of Histone H4.^31^ H4K20me3 has been primarily associated with heterochromatin formation but may have more complicated roles^32^ which could lead to changes in expression of many key osteoblastic genes during osteoblast differentiation. Ablation of *Setd4* in mouse bone marrow mesenchymal stem cells was reported to promote proliferation and decreased differentiation into a variety of cell types.^33^ Upregulation might be expected to have the opposite effect promoting differentiation into downstream cell types which is consistent with our findings here. Future functional studies are needed to elucidate the role of *STED4* in bone formation and what targets it might control during this process.

Furthermore, in addition to distant targets located from the BMD-associated loci, our analysis identified interactions with *RUNX1*, furthering confirming its role in the regulation of bone formation and changes in BMD. Our lab has pioneered the role of *RUNX1* in bone homeostasis, fracture repair and skeletal formation.^9–12^ Our most direct evidence for a function of *RUNX1* in skeletal homeostasis came from conditional deletion of *RUNX1* in myeloid cell populations, which resulted in increased bone resorption and decreased bone mass in young animals. Moreover, we established that *RUNX1* dose dependently regulates fracture callus formation and impairs soft callus development. Additionally, using *RUNX1* gain-of-function mice, we observed a sex-dependent effect where overexpression of *RUNX1* in female mice was shown to increase trabecular bone mass, most likely by delayed osteoclastogenesis and decreased bone resorption.^9^

We selected human iPSC derived mesenchymal stromal cells (iMSC) as a cellular model to study the interaction of BMD-associated variants with effector genes for bone formation. While acknowledging the involvement of other cell types, such as iMSC-derived osteoblasts and iPSC-derived osteoclasts in the determination of BMD, our focus was to unravel how this GWAS loci affect the bone formation in this parental cell types. To model the differentiation of human osteoblast in vitro, we generated mesenchymal stromal cells from iPSC lines derived from skin fibroblast using our established protocol.^26,34^ We assessed the expression of several mesenchymal marker genes using PCR and flowcytometric analysis in order to confirm that the iPSC derived cells molecularly resemble progenitor cells. As anticipated, these derived iMSCs expressed the mesenchymal stromal markers such as *TWIST1, RUNX1* and *COL1A1*, and do not express pluripotency marker such as *SOX2, NANOG, KLF4* etc. These iMSCs were further shown to express all the surface markers of mesenchymal stem cells as defined by minimal criteria of International Society of Cell and Gene Therapy (ISCT). Our analysis supports that iPSC derived iMSCs as an effective model system to study differentiation into osteoblast in vitro and bone formation in vivo.

To determine of role of these regulatory sequences on bone formation, we used an in vivo model of bone repair using murine calvarial defect. We used iMSCs derived from wild type and deletion mutants iPSCs (Δ1 and Δ 2) and implanted on parietal bone defects (2.3 mm, sub critical size) in athymic nude mice (NOD-SCID), which were used to prevent rejection of human cells. Continuous osteogenic induction was achieved by additional two dose of iMSCs implantation via transcutaneous injection at week 4 and week 8 into the defects. For sustained and prolonged delivery of iMSCs, we used synthetic PEG hydrogel as carrier. PEG hydrogels were made of four-arm PEG macromer that are end-functionalized by maleimide moieties (PEG-4MAL) to improve ligand binding and cross-linking. These hydrogels have been used extensively for targeted protein delivery because they are safe, elicit minimal inflammation, and have tunable properties.^30^ These hydrogels were previously used for encapsulation of bone marrow-derived mesenchymal stem cells (BM-MSCs) which were shown to exhibit enhanced osteo-reparative properties when implanted into murine bone defects.^35^ Using μCT analysis to quantify differences in bone volume (BV) within the cranial defect, our results demonstrate that iMSCs derived from homozygous deletion mutant 2 (Δ2-RUNX1-iMSCs, #27) showed significantly increased regenerated bone volume compared to wild-type iMSCs. Furthermore, H&E staining of these calvarial bone confirmed enhanced de-novo bone generation in Δ2-RUNX1-iMSCs treated group. These results suggest that deletion of regulatory sequences at BMD-GWAS loci near RUNX1 promoter may induce effector target gene (such as SETD4) which in turn promote osteogenic differentiation and enhanced bone formation in vivo.

To detect the presence of human cells at the repair sites, we performed immunofluorescence staining in calvarial sections using an anti-human vimentin antibody. Our results demonstrated the presence of immunoreactive human cells in calvarial defect area suggesting the involvement of human iMSCs in the bone regeneration at defect site. However, not all cells exhibited immunoreactivity with the human vimentin antibody. We interpret this as an indication that human iMSCs may not have persisted in the calvarial defect of the mice up to 12 weeks. Alternatively, or concurrently, both the implanted human cells and the host cells may have contributed to the repair of the defect site. Similar findings have been reported by several groups, where human cells were not detected after four weeks following the implantation of human adult MSCs in the calvarial bone of nude mice.^36,37^ Our findings are also consistent with previous studies in adult male nude mice, where engraftment of human adipose-derived stromal cells was observed primarily up to two weeks, with modest staining detectable up to four weeks post-implantation in critical-sized calvarial defects.^37^ We chose calvarial defect model as a regenerative model for in vivo assessment of osteogenic as well as osteo-inductive capacities of mutant iMSCs. This model allows us to evaluate these capacities in a native bone environment that facilitates interactions with native bone cells, vascularization, and mechanical forces, all of which are crucial for bone regeneration. Further studies are needed to identify the pure osteogenic subpopulation from these iMSCs, using recently reported skeletal osteogenic lineage markers.^38–40^ These studies will employ the kidney capsule implantation assay to assess the in vivo autonomous osteogenic potential of purified populations.

Our methodical approach, utilizing three-dimensional genomic data, functional genomics annotations, and genome-editing, has proven effective in identifying relevant target genes during osteoblast differentiation. Our findings show the BMD-associated variants at this locus demarcate potent chromatin-organizing sequences that influence expression of multiple genes over a large genomic region. Removal of these regions, while not the more subtle effects elicited by BMD-associated variants, revealed significant changes in gene expression that would likely have been difficult to detect otherwise. The identification of multiple putative effector genes underscores the need to empirically test what gene is regulated by loci identified by GWAS and not assume that the nearest gene is the most likely or only target.

## Materials and methods

### hiPSCs culture and maintenance

Human skin fibroblast-derived iPSCs (BD1-4, wild type) and their mutant (Δ) iPSCs were cultured using mTeSR™1 media (StemCell Technologies). The colonies were maintained in undifferentiated pluripotent state by culturing the iPSCs under feeder-free conditions in 6-well culture plate coated with 0.1% Geltrex® (Peprotech) as described previously.^26,27^ For routine culture and maintenance, these hiPSCs colonies were passaged after reaching 70% confluency by washing once with 1X-PBS and treating with ReLeSR™ reagent (StemCell Technologies) into new 6-well plate using mTeSR^TM^ 1 medium supplemented with 10 μmol/L Y-27632 Rock inhibitor (StemCell Technologies). Multiple clones from each of the mutant iPSC lines were used to ensure that our data are not clone specific. Pluripotency was determined by analyzing the expressions of canonical stemness genes (*SOX2, NANOG, OCT4, KLF4*) in each iPSC lines using SYBR®Green qPCR assay as previously reported.^41^

### Genomic editing of hiPSCs

Editing of hiPSCs was carried out by the University of Connecticut Cell and Genome Editing Core. The guide RNAs used for CRISPR/Cas9-mediated genome editing is shown in Fig. 3b. Two guide RNAs were chosen that flanked the region to be deleted using available guide RNA design tools. These guide RNA were cloned into PX459V2 using plasmid digested with bbs1 to allow for ligation of the guide RNA insert. Plasmid pSpCas9(BB)-2A-Puro (PX459) V2.0 was a gift from Feng Zhang (Addgene plasmid # 62988; RRID:Addgene 62988, http://n2t.net/addgene:62988).

Cells were grown on mitotically inactivated MEFs. 24 h prior to targeting, cells were treated with ROCKi (Y-27632). The following day, the cells were singularized with accutase and resuspended according to the protocol provided in the LONZA 4D nucleofection Primary P3 kit. 2 µgs of each CRISPR plasmid were added to the nucleofection solution. The cells were nucleofected using program CB-150. Cells were then plated on DR4 MEFs supplemented with ROCKi. The following day 0.5–1.0 ng/µL of puromycin and ROCKi was added to fresh media. This selection was continued for 48 h total to select cells transiently expressing the vector containing the guide RNA and Cas9 protein. ROCKi was kept on the cells until small colonies were observed. After this point the cell culture was continued with fresh media changes daily. After a total of 15 days colonies were isolated into 24 well plates. 4 days after plating in the 24 well plates, a few colonies from each well were isolated and DNA were extracted using the HOT SHOT method to prepare template for PCR. PCR was performed across the deletion region using Herculase II from Agilent. This screen used a primer upstream of the upstream CRISPR and a second primer downstream of the second CRISPR identified as 5F and 3R. A PCR product would indicate at least one allele was deleted from the clone, the primers in the unedited genome would be too far apart to produce a product near the expected size. Proceeding with the clones showing at least one allele deleted, a second reaction was run using primers found inside the region to be deleted. These primers are identified as intF and intR. Clones lacking this band showed that both alleles had the region deleted and the segment of DNA was no longer in the genome. Clones that positive for both the deletion band and the internal band were further screen by PCR using 5F and 5R to confirm the remaining allele was in the correct orientation.

### Immunofluorescence analysis for pluripotency markers in hiPSCs

Immunofluorescence staining in wild type and deletion mutants (ΔRUNX1-iPSCs) was performed as described previously.^26^ In brief, hiPSC lines were cultured in feeder-free condition in mTeSR^TM^1 medium. After reaching 80% confluency, hiPSC clones were with 4% paraformaldehyde, and permeabilized with 0.1% Triton X-100 in PBS. Pluripotent Stem Cell 4-Marker Immunocytochemistry Kit (Thermo Fischer Scientific) was used to stain the iPSC clones with antibody against SOX2 and TRA1-60 overnight at 4 °C followed by anti-rabbit IgG conjugated with Alexa Fluor™555, and Alexa Fluor-488. Nuclei were counterstained using DAPI with Fluoroshield Mounting (Abcam # ab104139). Images were acquired using Lionheart FX Automated Microscope (BioTek Instruments) as described previously.^9,22,26,42,43^

### Derivation of mesenchymal stromal cells from wild type and mutant ΔRunx1-iPSCs

The differentiation of osteoblasts from iPSCs necessitates an interim phase, denoted as mesenchymal progenitor cells or mesenchymal stem-like cells or medicinal sensing cells or mesenchymal stromal cells (iMSCs). To achieve the differentiation of iPSCs into iMSCs, we employed our established direct plating technique, outlined in our prior publications.^25–27,44^ Briefly, iPSC colony cell suspensions were generated through accutase treatment and subsequently seeded onto gelatin-coated culture plates using iMSC growth medium, comprising DMEM-High Glucose (Gibco), 10% defined fetal bovine serum (FBS; Hyclone), 1% nonessential amino acids (NEAA), 1X penicillin-streptomycin, and 5 ng/mL rhbFGF (Peprotech). After 2–3 passages, cells were cultured without any coating of plates where initial heterogeneous cultures adopted a homogenous, fibroblast-like morphology characteristic of iPSC-derived MSCs, referred to as iMSCs. For regular expansion, wild type and ΔRunx1-iPSCs derived MSCs (ΔRunx1-iMSCs) were plated at a density of 0.3–0.4 × 10^6^ cells per 10 cm culture dish and maintained in MSC growth media. The confirmation of MSC-like features involved the analysis of mesenchymal gene expression through a qPCR assay, and flow analysis of mesenchymal surface markers as previously outlined.^26,27^

### Flow analysis of mesenchymal stromal cells (iMSCs)

Immunophenotyping assessment for cell surface markers adhered to the criteria set by the International Society for Cell & Gene Therapy (ISCT) for the minimal standards of MSCs.^45^ Surface markers of MSCs were labeled and analyzed using anti-human antibodies against CD73, CD95, CD105, CD44, CD45, CD31, CD29, following a previously outlined method.^27,46^ Isotype-matched controls (IgG1-PE and IgG2b-FITC) were utilized to identify nonspecific fluorescence. BD FACSAria™, operated with FACS Diva software (Becton–Dickinson), was employed for cell acquisition. In each analysis, a minimum of 20 000 cells was acquired, and the data were analyzed using FlowJo Software, as detailed in previous work.^27,46^

### Osteogenic differentiation of iMSCs

To determine the role of regulatory sequences at BMD-GWAS SNPs near RUNX1 region on bone formation, we performed osteogenic differentiation of iMSCs derived from wild type and mutant iPSCs (ΔRUNX1-iMSCs) as described earlier.^47^ These iMSCs was cultured in 24-well plate at density of 10 000 cells per well. To prevent the peeling of cells during differentiation process, the culture plates were coated with rat type 1 collagen (10 µg/well, Sigma). The osteogenesis was induced by culturing the iMSCs in osteogenic medium consisting of alpha-MEM supplemented with 1 mmol/L sodium pyruvate, 0.1 μmol/L dexamethasone, 50 µg/mL ascorbic acid 2-phosphate, 10 mmol/L β-glycerophosphate, 10% FBS and 1X penicillin/streptomycin for 21 days. Medium was changed every 3 days and cells were harvested for RNA as described below and stained with Alizarin Red-S (40 mmol/L, pH 4.2) at 21 days. Briefly, cells were fixed in 10% neutral buffered formalin for 15 min and then stained with Alizarin Red-S for 5 min at room temperature. Dye was extracted using 10% acetic acid and absorbance quantified at 420 nm using a SpectraMax M2 plate reader (Molecular Devices).

### RNA isolation, reverse transcription, and qPCR analysis

RNA was isolated from hiPSCs, iMSCs and cells from osteogenic differentiation at day 7, 14 and 21 using TRIzol reagent (Invitrogen) as previously described.^26,48^ Reverse transcription was performed using high-capacity cDNA synthesis kit (Thermo Fisher) following manufacturer’s instructions. Quantitative polymerase chain reaction (qPCR) was performed using PowerUp™ SYBR® Green master mix (Applied Biosystems). All RT-qPCR experiments were performed with three biological replicates from each group and two technical replicates. Melt curve analysis was performed for specificity of primer and authenticity of amplicon. The mRNA expression of all the genes was normalized to β-actin mRNA and relative expression levels were calculated using the 2^−ΔΔCT^ method as described previously.^48^

### Calvarial bone defect model in NOD-SCID mice and implantation of iMSCs for osteogenic bone formation in vivo

All in vivo experiments were performed using procedural guidelines with appropriate approvals from the Institutional Animal Care and Use Committee of Emory University. Eight-week-old male NOD-SCID mice (001303-NOD.CB17-Prkdc<scid>/J) were purchase from Jackson laboratory. Sub critical size calvarial defect surgery was performed in parietal bone using our established method.^29^ Briefly, the surgery site was disinfected and then incisions were made using sterile surgical equipment to expose the parietal bones of the mice. Thereafter, 2.3 mm defects were created in the parietal bones using a variable speed drill (Aseptico (MicroNX), MAX-88ESP, CL1791023) and sterile circular knives.

PEG-4MAL hydrogels (20 μL) loaded with 100 000 iMSCs (Wild type BD-14 iMSCs, Δ1-RUNX1-iMSCs and Δ2-RUNX1-iMSCs) were placed within the defects created in parietal bones in the NOD-SCID mouse skulls as the first dose. A second dose of the hydrogels encapsulating iMSCs were administered as transcutaneous injections during week 4, and third dose was injected at week 8 to continue the bone regenerative effect of iMSCs. The skulls were harvested at week 12, fixed using 10% neutral buffered formalin (VWR, 89370-094), and bone formation was quantified using micro computed tomography (μCT) analysis.

### Hydrogel preparation

We prepared poly (ethylene glycol) (PEG)-based synthetic hydrogels incorporating cell adhesive peptides in two steps. First, maleimide end-functionalized 20 kD four-arm PEG macromer (PEG-4MAL, with >95% end-group substitution, Laysan Bio, 4ARM-PEG-MAL-20K), was reacted with a thiol-containing adhesive peptide GRGDSPC (Genscript, RP20283) in PBS with 20 mmol/L HEPES at pH 7.4 for 1 h. Then, the RGD-functionalized PEG-4MAL macromers were cross-linked in the presence of iMSCs into a hydrogel by addition of the dithiol protease-cleavable peptide cross-linker GPQ-W (GCRDGPQGIWGQDRCG) (New England Peptides, Inc, (NEP) Custom synthesized).^29^ The final gel formulation consisted of 4.0% wt/vol polymer and 1.0 mmol/L RGD.

### Micro computed tomography (μCT) analysis for quantifying bone regeneration

μCT analyses were conducted according to current guidelines for the assessment of bone volume within the defects created in mouse calvaria.^49^ Briefly, formalin-fixed skulls were positioned in the μCT tubes with the nose facing the bottom of the tube and imaged in a μCT 40 (Scanco Medical AG, Bassersdorf, Switzerland) using a 36 μm isotropic voxel size in all dimensions. Thereafter, using a consistent and pre-determined threshold of 55 kVp, 145 μA, 8 W, and 200 ms integration time for all measurements, three-dimensional (3D) reconstructions were created by stacking the regions of interest from ~600 two-dimensional (2D) slices consisting of the entire skull and then applying a gray-scale threshold of 150 and Gaussian noise filter (*σ* = 0.8, support = 1.0), a coronal reformatting was done. Thereafter, a circular region of interest encompassing the defect was selected for analysis consisting of transverse CT slices encompassing the entire defect and, new bone volume (BV) was calculated.

### Histological analysis and H&E staining

To see the structural changes in mice calvaria defect, formalin-fixed skulls used for µCT measurements were processed for histological assessment. Mice skulls were decalcified in Morse’s solution (10% sodium citrate and 22.5% formic acid) for 3 days, dehydrated with various grade of ethanol and then embedded in paraffin wax, and sectioned on a microtome to obtain sections at 5 μm thickness. These sections were then stained with H&E staining as described previously and imaged with brightfield microscopy.^50^

### Immunofluorescence analysis

To detect the presence of human cells at implantation site in mice calvarium, we performed immunofluorescence staining of paraffin sections as previously described.^26^ Briefly, we performed deparaffinization of 5 μm thick sections by incubating them for 45 min at 60 °C, followed by Xylene (2 times, 5 min each), and then sections were rehydrated using various grades of ethanol in a decreasing concentration (100%, 95% and 75%). The sections were washed with 1X PBS for 10 min, permeabilized with 1.0% Triton X-100 (in 1X PBS) for 10 min at room temperature and rinsed with 1X PBS (3 times, 5 min each). Antigen retrieval was performed by incubating these sections with 20 µg/mL of Proteinase K in 1X PBS for 20 min at 37 °C in a humidified chamber. Blocking was done using 1% Bovine Serum Albumin for 30 min at room temperature and sections were incubated with rabbit anti-human vimentin (Abcam, Cambridge, UK) for overnight at 4 °C. The slides were then washed three times with PBS washing buffer, incubated with secondary antibody Alexa Fluor 485 anti-rabbit (Thermo Fisher Scientific) for 45 min at 4 °C, and mounted with coverslips using mounting medium with 4′,6-diamidino-2-phenylindole (DAPI) (Vector, Burlingame, CA). Slide were imaged using fluorescence microscopy for the fluorescence detection.

### 4C-sequencing

Genomic viewpoint primers were designed using 4C-Seq primer db [http://compgenomics.weizmann.ac.il/tanay/?page_id=367] and primer sequences used for 4C viewpoints and indices were shown in Table 1. The database contains an in silico NlaIII/DpnII digest of the genome of interest, fragments with both NlaIII and DpnII cut site are aligned to the genome and assigned an ID (pp_id). The pp_id can be used to retrieve the sequence of the NlaIII and DpnII cut site to be used with addition of PE1 or PE2 adapter sequence. Primers were modified to a split design to permit re-use and multiplexing (Fig. S1). 4C-seq was performed according to van de Werken et al. ^51^ with some modifications.^51^ Cells or tissue were fixed as described in Cotney et al. ^52^ and nuclei isolated following homogenization with a dounce tissue grinder.^52^ Chromatin was digested with the combination NlaIII/DpnII. In summary, the isolated nuclei are permeabilized, chromatin digested with NlaIII, diluted and ligated with T4 DNA ligase, proteins are digested and ligated DNA is isolated, then digested with DpnII and ligated with T4 DNA ligase, then recovered by precipitation. The recovered DNA is cleaned up by Qiagen PCR purification column. 4C amplification is performed in two steps, PCR with genomic viewpoint primers plus PE1/PE2 adapter followed by PCR cleanup and PCR with primers containing desired indices for multiplexing.

**Table 1:** Primer sequences used for 4C viewpoints and indices

VP	Element	NlaIII-site primer	DpnII-site primer
VP1	*RUNX1* promoter 1	ACACTCTTTCCCTACACGACGCTCTTCCGATCTGACATCACTTAAGTCACATG	GTGACTGGAGTTCAGACGTGTGCTCTTCCGATCTGCTCCTGTTGTTATTTGTGG
VP2	rs13046645	ACACTCTTTCCCTACACGACGCTCTTCCGATCTTCCCTTTGTTATGGACCATG	GTGACTGGAGTTCAGACGTGTGCTCTTCCGATCTattggtttgctagagctgtc
VP3	rs2834676	ACACTCTTTCCCTACACGACGCTCTTCCGATCTAGGTGCCACAAGTTTTCATG	GTGACTGGAGTTCAGACGTGTGCTCTTCCGATCTGTCTTTCTTTCCCAACACTG
VP4	rs2834694	ACACTCTTTCCCTACACGACGCTCTTCCGATCTgctaggcactgttctccatg	GTGACTGGAGTTCAGACGTGTGCTCTTCCGATCTGTACCTGAGGGAGAAAGCTC
VP5	*RUNX1* promoter 2	ACACTCTTTCCCTACACGACGCTCTTCCGATCTAGGTGATTTGTACATACATG	GTGACTGGAGTTCAGACGTGTGCTCTTCCGATCTATTTGGAAGGTGTAGGAACA
VP6	*SETD4* promoter	ACACTCTTTCCCTACACGACGCTCTTCCGATCTTGGGGATGAGAAATGTCATG	GTGACTGGAGTTCAGACGTGTGCTCTTCCGATCTTATCTGGCTGTTCATCTTCC
index	Truseq_HT_D501	AATGATACGGCGACCACCGAGATCTACACTATAGCCTACACTCTTTCCCTACACGACGCTCTTCCGATCT	
index	Truseq_HT_D502	AATGATACGGCGACCACCGAGATCTACACATAGAGGCACACTCTTTCCCTACACGACGCTCTTCCGATCT	
index	Truseq_HT_D503	AATGATACGGCGACCACCGAGATCTACACCCTATCCTACACTCTTTCCCTACACGACGCTCTTCCGATCT	
index	Truseq_HT_D504	AATGATACGGCGACCACCGAGATCTACACGGCTCTGAACACTCTTTCCCTACACGACGCTCTTCCGATCT	
index	TruSeq_HT_revcom_D701	CAAGCAGAAGACGGCATACGAGATCGAGTAATGTGACTGGAGTTCAGACGTGTGCTCTTCCGATCT	
index	TruSeq_HT_revcom_D702	CAAGCAGAAGACGGCATACGAGATTCTCCGGAGTGACTGGAGTTCAGACGTGTGCTCTTCCGATCT	
index	TruSeq_HT_revcom_D703	CAAGCAGAAGACGGCATACGAGATAATGAGCGGTGACTGGAGTTCAGACGTGTGCTCTTCCGATCT	
index	TruSeq_HT_revcom_D704	CAAGCAGAAGACGGCATACGAGATGGAATCTCGTGACTGGAGTTCAGACGTGTGCTCTTCCGATCT	

4C-seq libraries were sequenced using the NextSeq550 (Illumina, USA). Fastq files were demultiplexed by barcode yielding Fastq files for each tissue replicate. Fastq files were further demultiplexed by viewpoint using Trimmomatic.^53^ Trimmed reads were aligned using bowtie2.^54^ The location of the viewpoint and sequenced interacting fragment are denoted with thick bars. Significant interactions in wild type (WT) and deletion cell lines (Δ1 and Δ2) were assessed using PeakC.^19^ The significant interactions are represented using the UCSC interact track format. Scripts used for 4C-seq data processing are available at github.com/awilderman/RUNX1-4C.

### Use of publicly available HiC data

Sequence data from HiC experiments performed on H1 hESC-derived MSCs^34^ were downloaded from SRA (SRR1030739-SRR1030744) and merged following quality control analysis of the fastq files. Data was initially processed using HiC-Pro v.2.10.0^55^ visualization and prediction of TADs were done using HiCExplorer v.3.7.^56^ Scripts used for processing and visualization of HiC data are available at github.com/awilderman/RUNX1-4C.

### Statistical analysis

Data are expressed as mean ± SEM of at least three independent experiments. All experiments represent biological replicates and were repeated at least three times unless otherwise stated. Biological replicates are samples derived from separate sources, such as different clones of iPSCs and iMSCs. Statistical comparisons between two groups (WT- vs Δ1-RUNX1/Δ2- RUNX1) were performed using a two-tailed Student’s t-test for comparing two groups using GraphPad Prism. However, comparison between three or more groups (WT- vs Δ1-RUNX1 vs Δ2-RUNX1) were performed using one-way ANOVA followed by posthoc analysis using Tukey’s HSD. Significance was set at *P* < 0.05.

## Supplementary information

Fig. S1

Fig. S2

Fig. S3

Fig. S4

Legends to Supplementary Figures

## Data Availability

The 4C-seq data generated in the current study will be made available upon acceptance in public domain.
