# Supplementary figures and images for: Enhanced osteogenic potential of iPSC-derived mesenchymal progenitor cells following genome editing of GWAS variants in the RUNX1 gene
Source: Bone Res. 2024 Dec 6;12:70. doi: 10.1038/s41413-024-00369-x (PMC11624199; doi:10.1038/s41413-024-00369-x)

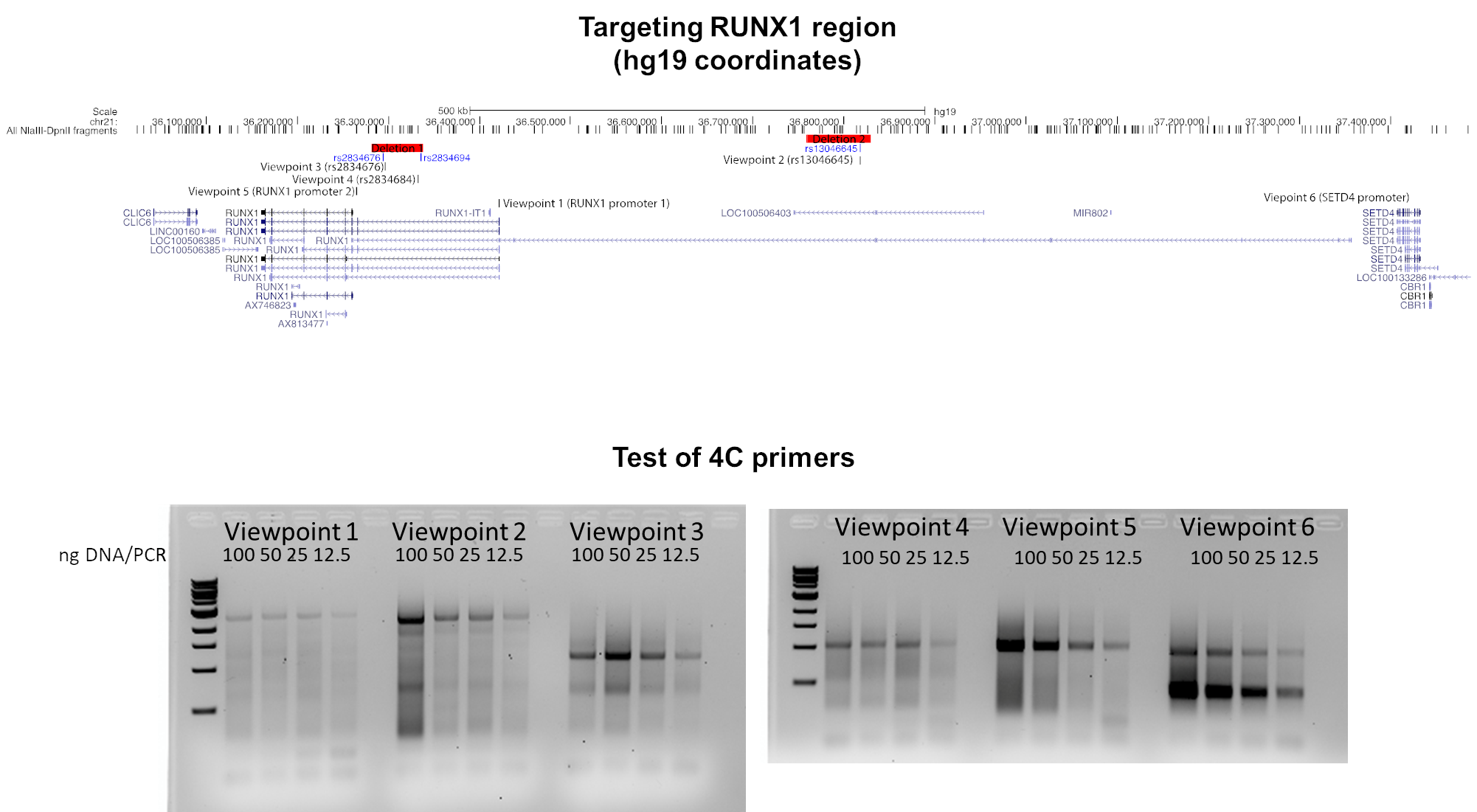

Supplement: Supplementary file 1 — Fig. S1 [file 41413_2024_369_MOESM1_ESM.tif]

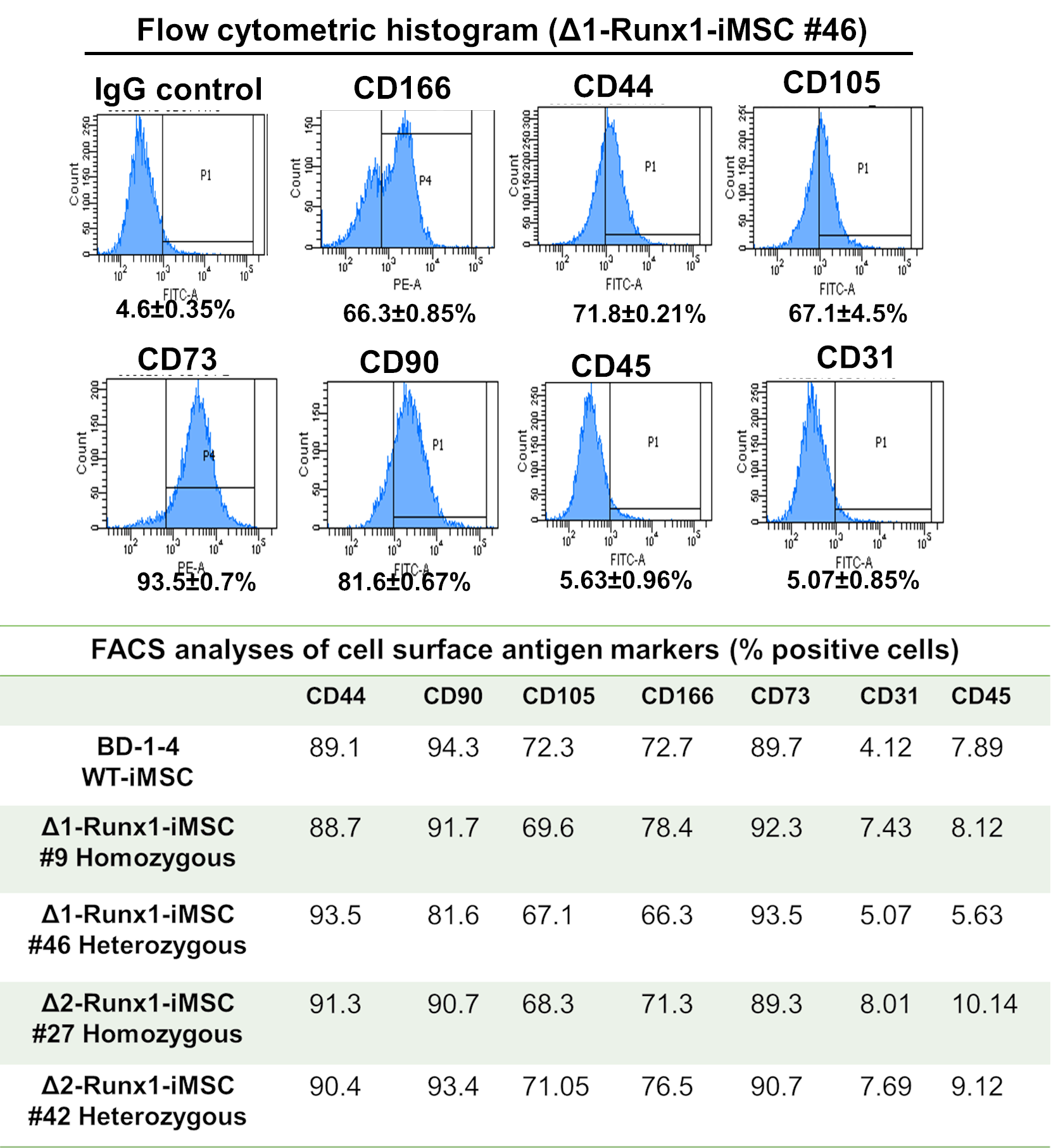

Supplement: Supplementary file 2 — Fig. S2 [file 41413_2024_369_MOESM2_ESM.tif]

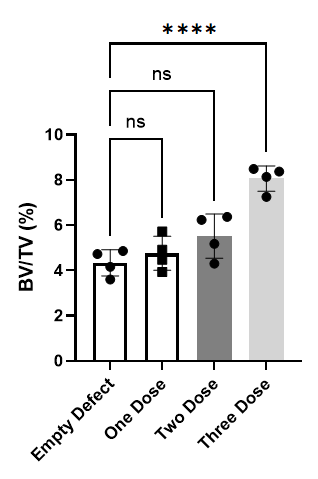

Supplement: Supplementary file 3 — Fig. S3 [file 41413_2024_369_MOESM3_ESM.tif]

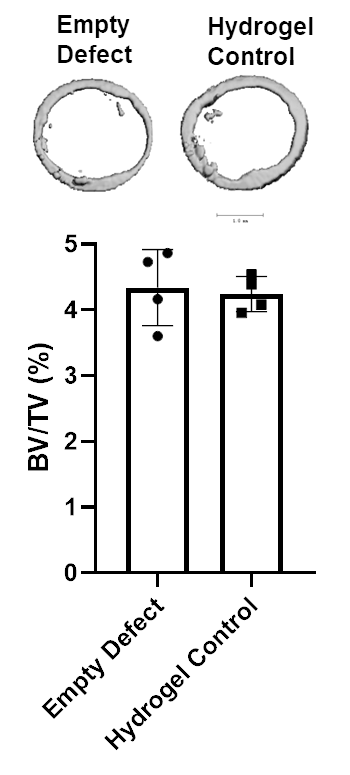

Supplement: Supplementary file 4 — Fig. S4 [file 41413_2024_369_MOESM4_ESM.tif]
