# Supplementary material for: Enhanced osteogenic potential of iPSC-derived mesenchymal progenitor cells following genome editing of GWAS variants in the RUNX1 gene
Source: Bone Res. 2024 Dec 6;12:70. doi: 10.1038/s41413-024-00369-x (PMC11624199; doi:10.1038/s41413-024-00369-x)
Supplement: Supplementary file 5 — Legends to Supplementary Figures [file 41413_2024_369_MOESM5_ESM.docx]

**LEGENDS TO SUPPLEMENTARY FIGURES:**

**Figure S1:** Genomic viewpoint of primers used 4C-Sequencing. Primers multiplexing standardization are shown.

**Figure S2:** Expression of surface antigens in Δ1-*RUNX1*-iMSCs by flow analysis. Representative flow cytometric histogram showing Δ1-*RUNX1*-iMSCs (#46) express markers associated with the mesenchymal phenotype (positive for CD44, CD73, CD90, CD105, and CD166; negative for CD31, and CD45) (n=3); Comparative flow cytometry analyses of wild type (BD1-4) and Δ1- *RUNX1*-iMSCs (#9, #46) and Δ2-*RUNX1*-iMSCs (#27, #42) showing similar cell surface expression profiles. Results from one representative experiment (n=3).

**Figure S3:** Pilot study showing efficacy of single vs multi-injection of iMSCs in a PEG hydrogel stimulates bone regeneration in a calvarial defect model. Three groups of mice with 2.3 mm calvarial defect were implanted with single or two or three dose of iMSCs encapsulated in 4% PEG-MAL hydrogels and after eight weeks, we quantified differences in regenerated bone volume within the defect and compared them between experimental groups by μCT analysis. (N=5), Data are presented as mean ± SD.

**Figure S4:** Pilot study showing effect PEG hydrogel on bone regeneration in a calvarial bone defect mouse model. No cells (Empty Defect) or Hydrogel alone groups 4% PEG-MAL hydrogels implanted into parietal bones of 8-week-old NOD SCID mice. After eight weeks, μCT analysis was done to quantify regenerated bone volume within the defect. Data are presented as mean ± SD, (N=5).
